# Erlotinib or Gefitinib for Treating Advanced Epidermal Growth Factor Receptor Mutation–Positive Lung Cancer in Aotearoa New Zealand: Protocol for a National Whole-of-Patient-Population Retrospective Cohort Study and Results of a Validation Substudy

**DOI:** 10.2196/51381

**Published:** 2024-07-02

**Authors:** Phyu Sin Aye, Joanne Barnes, George Laking, Laird Cameron, Malcolm Anderson, Brendan Luey, Stephen Delany, Dean Harris, Blair McLaren, Elliott Brenman, Jayden Wong, Ross Lawrenson, Michael Arendse, Sandar Tin Tin, Mark Elwood, Philip Hope, Mark James McKeage

**Affiliations:** 1 Department of Pharmacology and Clinical Pharmacology University of Auckland Auckland New Zealand; 2 School of Pharmacy University of Auckland Auckland New Zealand; 3 Te Aka Mātauranga Matepukupuku Centre for Cancer Research University of Auckland Auckland New Zealand; 4 Department of Medical Oncology, Te Pūriri o Te Ora Regional Cancer and Blood Service Te Whatu Ora Health New Zealand Auckland City Hospital Auckland New Zealand; 5 Department of Medical Oncology Te Whatu Ora Health New Zealand Te Pae Hauuora o Ruahine o Tararua Palmerston North Hospital Palmerston North New Zealand; 6 Wellington Blood and Cancer Centre Te Whatu Ora Health New Zealand Capital, Coast and Hutt Valley Wellington Hospital Wellington New Zealand; 7 Department of Oncology Te Whatu Ora Health New Zealand Nelson Marlborough Nelson Hospital Nelson New Zealand; 8 Oncology Service Te Whatu Ora – Waitaha Canterbury Christchurch Hospital Christchurch New Zealand; 9 Southern Blood and Cancer Service Te Whatu Ora Southern Dunedin Hospital Dunedin New Zealand; 10 Cancer and Haematology Services Te Whatu Ora Health New Zealand Haora a Toi Bay of Plenty Tauranga Hospital Tauranga New Zealand; 11 Cancer Services Te Whatu Ora Health New Zealand Waikato Waikato Hospital Hamilton New Zealand; 12 Medical Research Centre University of Waikato Hamilton New Zealand; 13 Department of Pathology Te Whatu Ora Health New Zealand Waikato Waikato Hospital Hamilton New Zealand; 14 Department of Epidemiology and Biostatistics University of Auckland Auckland New Zealand; 15 Lung Foundation New Zealand Manukau Auckland New Zealand; 16 Auckland Cancer Society Research Centre University of Auckland Auckland New Zealand

**Keywords:** epidermal growth factor receptor, erlotinib, gefitinib, lung cancer, retrospective cohort, study protocol, validation

## Abstract

**Background:**

Starting in 2010, the epidermal growth factor receptor (EGFR) kinase inhibitors erlotinib and gefitinib were introduced into routine use in Aotearoa New Zealand (NZ) for treating advanced lung cancer, but their impact in this setting is unknown.

**Objective:**

The study described in this protocol aims to understand the effectiveness and safety of these new personalized lung cancer treatments and the contributions made by concomitant medicines and other factors to adverse outcomes in the general NZ patient population. A substudy aimed to validate national electronic health databases as the data source and the methods for determining patient eligibility and identifying outcomes and variables.

**Methods:**

This study will include all NZ patients with advanced EGFR mutation–positive lung cancer who were first dispensed erlotinib or gefitinib before October 1, 2020, and followed until death or for at least 1 year. Routinely collected health administrative and clinical data will be collated from national electronic cancer registration, hospital discharge, mortality registration, and pharmaceutical dispensing databases by deterministic data linkage using National Health Index numbers. The primary effectiveness and safety outcomes will be time to treatment discontinuation and serious adverse events, respectively. The primary variable will be high-risk concomitant medicines use with erlotinib or gefitinib. For the validation substudy (n=100), data from clinical records were compared to those from national electronic health databases and analyzed by agreement analysis for categorical data and by paired 2-tailed *t* tests for numerical data.

**Results:**

In the validation substudy, national electronic health databases and clinical records agreed in determining patient eligibility and for identifying serious adverse events, high-risk concomitant medicines use, and other categorical data with overall agreement and κ statistic of >90% and >0.8000, respectively; for example, for the determination of patient eligibility, the comparison of proxy and standard eligibility criteria applied to national electronic health databases and clinical records, respectively, showed overall agreement and κ statistic of 96% and 0.8936, respectively. Dates for estimating time to treatment discontinuation and other numerical variables and outcomes showed small differences, mostly with nonsignificant *P* values and 95% CIs overlapping with zero difference; for example, for the dates of the first dispensing of erlotinib or gefitinib, national electronic health databases and clinical records differed on average by approximately 4 days with a nonsignificant *P* value of .33 and 95% CIs overlapping with zero difference. As of May 2024, the main study is ongoing.

**Conclusions:**

A protocol is presented for a national whole-of-patient-population retrospective cohort study designed to describe the safety and effectiveness of erlotinib and gefitinib during their first decade of routine use in NZ for treating EGFR mutation–positive lung cancer. The validation substudy demonstrated the feasibility and validity of using national electronic health databases and the methods for determining patient eligibility and identifying the study outcomes and variables proposed in the study protocol.

**Trial Registration:**

Australian New Zealand Clinical Trials Registry ACTRN12615000998549; https://www.anzctr.org.au/Trial/Registration/TrialReview.aspx?id=368928

**International Registered Report Identifier (IRRID):**

DERR1-10.2196/51381

## Introduction

### Background

Over the last 20 years, advances in the understanding of the molecular basis of lung cancer have led to new diagnostic and treatment practices being introduced into routine care [1], but their everyday impact in general patient populations is undefined. Such improvements in lung cancer care have been an important priority because lung cancer is a major cause of death globally [2] and a significant contributor to ethnicity-related health inequity in Aotearoa New Zealand (NZ) [3] and elsewhere. Starting in 2010, the epidermal growth factor receptor (EGFR) kinase inhibitor drugs erlotinib and gefitinib were introduced into routine clinical use in NZ for the treatment of advanced lung cancer [4]. Before their introduction, clinical trials of erlotinib and gefitinib had demonstrated substantial therapeutic benefits in patients with EGFR mutation–positive lung cancer, with >50% reduction in the risk of disease progression in pooled analyses of randomized trials [5]. These clinical trials also highlighted major safety concerns with erlotinib and gefitinib due to serious adverse drug reactions [6], including interstitial lung disease, renal failure, hepatotoxicity, severe diarrhea, gastrointestinal perforation, and skin and ocular disorders [7,8]. Regulatory studies [9,10] also alerted to the potential interaction of erlotinib and gefitinib with other medicines, expected to lead to adverse outcomes from treatment. Some medicine interactions were expected to reduce lung cancer treatment effectiveness by reducing systemic exposure to erlotinib or gefitinib from their concurrent use with gastric acid suppressants [11,12] or cytochrome P450 enzyme inducers [13,14]. Other medicine interactions were expected to increase serious adverse events by increasing erlotinib or gefitinib systemic exposure from their concurrent use with cytochrome P450 enzyme inhibitors [14,15]. Other medicine interactions were expected to increase the risk of gastrointestinal perforation from concurrent use with nonsteroidal anti-inflammatory drugs or systemic corticosteroids or bleeding from prolongation of the international normalized ratio from concurrent use with coumarin anticoagulants [16,17]. Despite these expectations, the effectiveness and safety of these new personalized lung cancer treatments in the general population of NZ patients has not yet been described, and the contributions made by concomitant medicines and other factors to the occurrence of adverse outcomes remain to be defined in this setting of routine care. As clinical trials underreport harm from anticancer drugs [18] and assess their efficacy under ideal rather than everyday circumstances [19], large-scale observational studies such as the one described in this protocol are required to fully understand the safety and effectiveness of cancer treatment in real-world settings. Recent reports illustrate how real-world studies of new personalized lung cancer treatments can be informative about their effectiveness and safety in general patient populations [20] and unrepresented patient groups [21] as well as the impacts of concomitant medicines [22].

Studies using routinely collected health administrative and clinical data can provide insights into the everyday impacts of new treatment pathways, but their data sources and study methodology first need to be validated [23]. In NZ, cancer registration, hospital discharge, mortality registration, and pharmaceutical dispensing data are collected in national electronic health databases [24], thereby providing potentially useful sources of information for studies of the impacts of routine cancer care. However, bias may be introduced from errors in data recording or in the methods used for identifying the cases and health outcomes of interest, which may limit the validity of study findings. This potential for bias may be controlled in part by quantifying errors inherent in study methodology and data sources by comparison against a reference standard, such as clinical records [25].

### Objectives

The purpose of this paper is to present the study protocol and results of a validation substudy for a planned retrospective cohort study of the safety and effectiveness of erlotinib and gefitinib during the first decade of their use in NZ for treating advanced EGFR mutation–positive lung cancer. The overall objective of the study described in this protocol is to understand the effectiveness and safety of new personalized treatments in the general population of NZ patients with lung cancer and the contributions made by concomitant medicines and other factors to adverse outcomes in this setting of routine care. The study protocol and results of the validation substudy are detailed in the following sections.

The specific aims of the study are to (1) determine the effectiveness and safety of erlotinib and gefitinib for the treatment of EGFR mutation–positive lung cancer in the entire cohort of all patients treated in NZ over a 10-year period, (2) identify serious adverse events and assess their expected association with erlotinib and gefitinib in these patients, (3) determine the prevalence and patterns of the concomitant use of other medicines with erlotinib or gefitinib in these patients, and (4) assess whether the combined use of other medicines was associated with serious adverse events or reduced lung cancer treatment effectiveness in these patients treated with erlotinib or gefitinib.

## Methods

### Study Design

This study will be a national, population-based, observational, data-linkage, retrospective cohort study that will analyze routinely collected administrative and clinical electronic health data. The study was registered on the Australian New Zealand Clinical Trials Registry (ACTRN12615000998549).

### Study Group

This study will have a whole-of-population sample comprising a single group of patients treated with erlotinib or gefitinib for EGFR mutation–positive lung cancer who were first dispensed erlotinib or gefitinib before October 1, 2020, and followed thereafter until death or for at least 1 year.

### Setting

NZ had a resident population of approximately 4.4 to 5.1 million people during the period of the planned retrospective study. NZ residents were eligible for state-funded health care, including prescription medicines whose state subsidies were managed by the NZ government agency Te Pātaka Whaioranga–Pharmac [26]. From October 1, 2010, to December 31, 2013, Te Pātaka Whaioranga–Pharmac fully funded erlotinib in NZ as a second-line treatment for advanced non–small cell lung cancer without any requirement for EGFR mutation testing. From August 1, 2012, Te Pātaka Whaioranga–Pharmac fully funded gefitinib in NZ as a first-line treatment for advanced EGFR mutation–positive non–small cell lung cancer. On May 1, 2013, the National Health Committee of the NZ Ministry of Health issued recommendations for EGFR mutation testing in NZ, including that all patients with non–small cell lung cancer should be tested at diagnosis as part of standard pathology processes, except those confidently diagnosed with squamous carcinoma. From January 1, 2014, Te Pātaka Whaioranga–Pharmac restricted funding for erlotinib in NZ to the treatment of advanced EGFR mutation–positive non–small cell lung cancer.

### Eligibility Criteria

#### Inclusion Criteria

Patients will be eligible for inclusion in the retrospective cohort if they were (1) diagnosed with EGFR mutation–positive lung cancer; (2) dispensed erlotinib and gefitinib first before October 1, 2020; and (3) followed thereafter until death or for at least 1 year.

#### Exclusion Criteria

Patients will be excluded from the retrospective cohort if they had (1) a dispensing of erlotinib or gefitinib before positive EGFR mutation test results became required for state subsidies for erlotinib (starting January 1, 2014) and gefitinib (starting August 1, 2012) in NZ, (2) no notification of a diagnosis of nonsquamous non–small cell lung cancer in the NZ Cancer Registry, or (3) an unactionable or unknown EGFR mutation subtype.

#### Proxy Criteria for the Initial Assessment of Patient Eligibility

As EGFR mutation test results were not systematically recorded in national electronic health databases, proxy criteria were developed for identifying an initial pool of potentially eligible patients. According to these proxy criteria, patients will be potentially eligible for inclusion if they were first dispensed gefitinib on or after August 1, 2012, or erlotinib on or after January 1, 2014, which was when positive EGFR mutation test results first became required for access to state-subsidized erlotinib and gefitinib in NZ. Patients dispensed erlotinib before January 1, 2014, or gefitinib before August 1, 2012, will be excluded because of uncertainty about their EGFR mutation status at the time of commencing treatment with erlotinib or gefitinib. A further assessment of potentially eligible patients will be undertaken according to the aforementioned inclusion and exclusion criteria. The validity of these proxy criteria for an initial evaluation of patient eligibility was assessed in the validation substudy (Multimedia Appendix 1).

### Data Sources and Linkage

**Figure 1:**
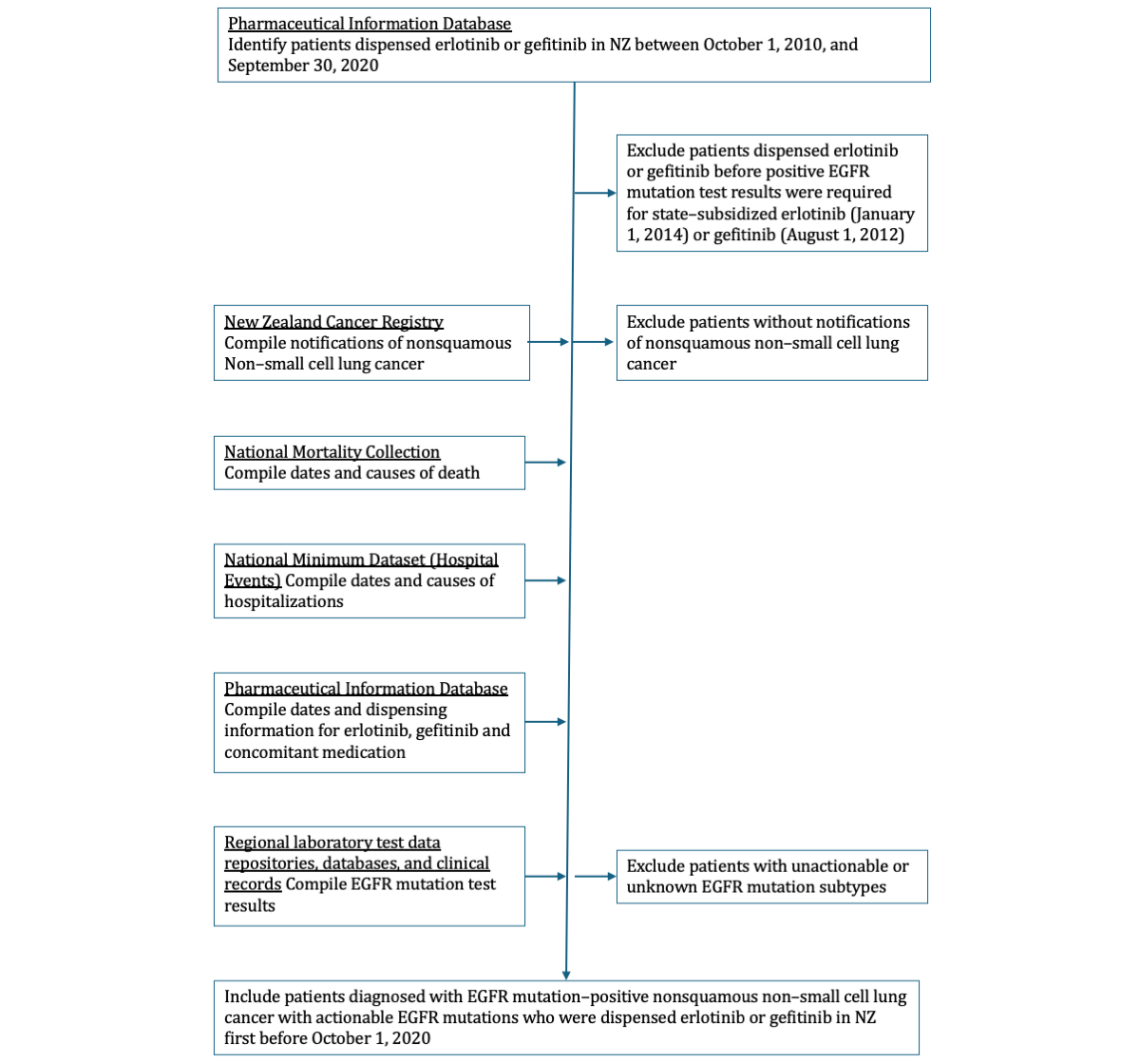
Flow diagram of the assembly of a retrospective cohort and compilation of data from national electronic health databases for a study of the safety and effectiveness of erlotinib and gefitinib for treating advanced epidermal growth factor receptor (EGFR) mutation–positive lung cancer in Aotearoa New Zealand (NZ).

Eligible patients will be identified first in national electronic dispensing records of erlotinib and gefitinib (Figure 1). Subsequently, the data required for the study will be compiled from these dispensing records and other sources (Figure 1). Use will be made of routinely collected health administrative and clinical electronic data sourced from various national electronic health databases, including (1) the New Zealand Cancer Registry, which is a population-based register of all legally mandated notifications of cancer diagnoses and deaths in NZ and includes all cancer types except nonmelanoma skin cancer; (2) the Pharmaceutical Information Database, which contains payment and claims information from pharmacists for state-subsidized community pharmaceutical dispensings in NZ; (3) the National Mortality Collection, which contains information on all deaths registered in NZ and includes the dates and underlying cause of death; and (4) National Minimum Dataset (Hospital Events), which is a national collection of public and private hospital discharge information for day patients and inpatients in NZ. Individual-level data on eligible cohort participants will be compiled from these various national electronic health databases by deterministic data linkage using each participant’s unique National Health Index number. Additional data on eligible cohort participants may be sourced from regional data repositories or clinical records, such as EGFR mutation subtype and testing methodology.

### Outcomes

#### Primary Effectiveness Outcome

The primary effectiveness outcome measure will be time to treatment discontinuation with erlotinib or gefitinib. Prescriber guidelines [7,8] recommend uninterrupted daily treatment*,* with erlotinib and gefitinib continued for as long as it is safe and tolerable, and the patient is responding. In clinical trials of EGFR kinase inhibitor drugs for the treatment of advanced EGFR mutation–positive lung cancer, time to treatment discontinuation correlated well with progression*-*free survival [27], which is an important and common end point used in oncology trials to measure the efficacy of a drug. Time to treatment discontinuation is also less impacted by subsequent treatments and other factors that confound the effects of study treatment on overall survival. For this study, time to discontinuation of treatment with erlotinib or gefitinib will be defined as the duration between the dates of first dispensing and last treatment with erlotinib or gefitinib. The date of last treatment with erlotinib or gefitinib will be calculated by adding the number of days’ treatment with erlotinib or gefitinib dispensed at the last dispensing to the date of this last dispensing, except when death occurred before the calculated date of last treatment*,* in which case the date of last treatment will be the date of death. Participants with no known dates of last treatment or death will be censored at the date of their last known treatment. The validity of national electronic health databases as the source of key dates determining time to treatment discontinuation was assessed in the validation substudy (Multimedia Appendix 2).

#### Primary Safety Outcome

The primary safety outcome measure will be serious adverse events. For this study, serious adverse events will be defined as death or hospitalization during or within 30 days of stopping treatment with erlotinib or gefitinib, when the primary cause of death or hospitalization was non–cancer related. Cancer-related hospitalizations and deaths will be excluded from serious adverse events because lung cancer progression and death are expected outcomes during palliative treatment of advanced lung cancer with erlotinib or gefitinib. Cancer-related hospitalizations and deaths will be identified from the International Classification of Diseases, Tenth Revision (ICD-10), codes (C00-D48) for the main cause of hospitalization or death in national electronic discharge summary and mortality databases. The validity of national electronic health databases for identifying serious adverse events was assessed in the validation substudy (Multimedia Appendix 1).

#### Secondary Effectiveness Outcome

The secondary effectiveness outcome measure will be overall survival. Overall survival will be defined as the duration between the dates of the first dispensing of erlotinib or gefitinib and death from any cause. Participants with no known date of death will be censored at the date of their last known pharmaceutical dispensing or hospital discharge. The validity of national electronic health databases as the source of dates determining overall survival was assessed in the validation substudy (Multimedia Appendix 2).

#### Secondary Safety Outcomes

A secondary safety outcome measure will be interruption, reduction, or substitution of treatment with erlotinib or gefitinib. Prescriber guidelines recommend administering erlotinib 150 mg or gefitinib 250 mg once daily orally continuously without interruption except in the event of serious adverse drug reactions when erlotinib or gefitinib are recommended to be withheld, dose reduced, discontinued, or substituted by another treatment [7,8]. Such changes in dosing may signal significant safety concerns. Treatment interruption will be defined as when the total number of days of treatment with erlotinib and gefitinib dispensed was less, by ≥7 days, than the time to treatment discontinuation defined from the start and end dates of treatment with erlotinib or gefitinib. Treatment reduction will be defined as when the daily dose of treatment was reduced and substitution when erlotinib was changed to gefitinib, or vice versa. The validity of national electronic health databases for identifying patients with interruptions, reductions, or substitutions of treatment with erlotinib or gefitinib was assessed in the validation substudy (Multimedia Appendix 1).

Another secondary safety outcome will be serious adverse drug reactions. Serious adverse drug reactions are a subgroup of serious adverse events (primary safety outcome measure) known to be associated with erlotinib or gefitinib. Adverse drug reactions known to be associated with erlotinib or gefitinib monotherapy are listed in Textbox 1. They were identified in the warnings and precautions sections of the latest Food and Drug Administration drug label for erlotinib and gefitinib or as recorded elsewhere within their latest Food and Drug Administration drug label as being consistently increased compared to placebo in controlled trials of erlotinib or gefitinib monotherapy [7,8]. For this study, serious adverse drug reactions will be defined as those known to be associated with erlotinib or gefitinib monotherapy (Textbox 1) that resulted in death or hospitalization during or within 30 days of stopping treatment with erlotinib or gefitinib. Patients experiencing serious adverse drug reactions will be identified from the ICD-10 codes for the main cause of hospitalization or death during the relevant time period in national electronic discharge summary and mortality databases. The ICD-10 codes corresponding to adverse drug reactions known to be associated with erlotinib and gefitinib are shown in Textbox 1. The validity of national electronic health databases for identifying patients with serious adverse drug reactions to erlotinib or gefitinib was assessed in the validation substudy (Multimedia Appendix 1).

**Adverse drug reactions known to be associated with erlotinib or gefitinib, with their corresponding International Classification of Diseases, Tenth Revision (ICD-10), codes, compiled for a national retrospective cohort study of the safety and effectiveness of erlotinib and gefitinib for treating advanced epidermal growth factor receptor mutation–positive lung cancer in Aotearoa New Zealand.**

**Adverse drug reactions known to be associated with erlotinib or gefitinib and the corresponding ICD-10 codes**

- Decreased appetite: R63.0
- Dehydration: E86
- Diarrhea: A09.9, K52.1, and K52.9
- Dyspnea: R06.0
- Electrolyte abnormalities: E87.1-E87.5, E87.6, and E87.8
- Gastrointestinal perforation: K22.3, K25.1, K25.2, K25.5, K25.6, K26.1, K26.2, K26.5, K26.6, K27.1, K27.2, K27.5, K27.6, K28.1, K28.2, K28.5, K28.6, K63.1, K57.0, K57.2, K57.4, K57.8, K82.2, and K83.2
- Hemorrhage in patients on warfarin: D68.3
- Hepatotoxicity: R94.5, K71.0-K71.9, and K72.0-K72.9
- Interstitial lung disease: J70.2-J70.4 and J84.9
- Nausea and vomiting: R11
- Ocular disorders: H00.0-H22.8
- Renal failure: N14.1, N14.2, M14.4, N17.0-N17.9, N18.1-N18.9, and N19
- Skin and subcutaneous tissue disorders: L00-L99.8
- Stomatitis: K12.0-K13.7

#### Other Secondary Outcomes

Another secondary effectiveness outcome measure will be lung cancer–specific survival, measured from the start date of treatment with erlotinib or gefitinib until the date of death from lung cancer. Participants who died of causes other than lung cancer will be censored at their date of death. Participants with no known date of death will be censored at the date of their last known pharmaceutical dispensing or hospital discharge. Another secondary safety outcome measure will be non–cancer-related mortality, defined as when the primary cause of death was non–cancer related. For the analysis of non–cancer-related mortality, participants whose death was caused by cancer will be censored at their date of death. Participants with no known date of death will be censored at the date of their last known pharmaceutical dispensing or hospital discharge. As the primary cause of death is not systematically recorded in clinical records, these secondary outcome measures were not formally validated.

### Variables

#### Primary Variable

The primary variable will be the use of high-risk concomitant medicines during treatment with erlotinib or gefitinib. High-risk concomitant medicines will be those listed in Stockley’s Interactions Checker [28] with alerts to potential interaction with erlotinib or gefitinib, excluding natural health products, recreational drugs, foods, nonprescription medicines, medicines not used in NZ, medicines whose interaction is expected to result in only a mild or no effect, and topical formulations (Therapeutic group 2 or 3: ID numbers 105, 1011, 1019, 1033, 2810, 2824, 2851, 3103 and 10404; Textbox 2). High-risk concomitant medicines will be categorized according to whether their potential interaction is expected to (1) reduce systemic exposure to erlotinib or gefitinib, (2) increase systemic exposure to erlotinib or gefitinib, (3) increase the risk of gastrointestinal perforation, (4) increase international normalized ratio and bleeding risk, (5) increase interacting drug exposure and toxicity, or (6) alter the distribution and excretion of erlotinib or gefitinib (Textbox 2). Concurrent use will be defined as the dispensing of high-risk concomitant medicines at any time during treatment with erlotinib or gefitinib. The validity of national electronic health databases for identifying concurrent use of high-risk concomitant medicines was assessed in the validation substudy (Multimedia Appendix 1).

**High-risk concomitant medicines, categorized by their expected adverse outcome from potential interaction with erlotinib or gefitinib, compiled for a national retrospective cohort study of the safety and effectiveness of erlotinib and gefitinib for treating advanced epidermal growth factor receptor mutation–positive lung cancer in Aotearoa New Zealand.**

**Expected adverse outcome from potential drug interaction and high-risk concomitant medicines**

- Reduced exposure to erlotinib or gefitinib: antacids, bosentan, calcium carbonate, carbamazepine, cimetidine, dabrafenib, efavirenz, enzalutamide, famotidine, lansoprazole, mitotane, nevirapine, omeprazole, oxcarbazepine, pantoprazole, phenobarbital, phenytoin, primidone, ranitidine, rifabutin (erlotinib only), rifampicin, sodium bicarbonate, sorafenib, and temozolomide (erlotinib only)
- Increased exposure to erlotinib or gefitinib: aprepitant, atazanavir, bupropion (gefitinib only), cinacalcet (gefitinib only), ciprofloxacin (erlotinib only), clarithromycin, cobicistat, ethinylestradiol (erlotinib only), crizotinib, darunavir, diltiazem, erythromycin, fluconazole, fluoxetine (gefitinib only), imatinib, itraconazole, ketoconazole, lopinavir, methoxsalen (erlotinib only), mexiletine (erlotinib only), neratinib (erlotinib only), nilotinib, paclitaxel (gefitinib only), paroxetine (gefitinib only), posaconazole, ritonavir, terbinafine (gefitinib only), verapamil, and voriconazole
- Increased risk of gastrointestinal perforation (considered a class effect but specified only for erlotinib and not for all systemic corticosteroids in Stockley’s Interactions Checker): aspirin, betamethasone, budesonide, celecoxib, cortisone, dexamethasone, diclofenac, docetaxel, etoricoxib, fludrocortisone, hydrocortisone, ibuprofen, indometacin, ketoprofen, ketorolac, mefenamic acid, meloxicam, methylprednisolone, naproxen, paclitaxel, parecoxib, prednisolone, prednisone, sulindac, tenoxicam, and tiaprofenic acid
- Increased international normalized ratio and bleeding risk: acenocoumarol, phenindione, and warfarin
- Increased interacting drug exposure and toxicity: alprazolam (erlotinib only), etoposide (erlotinib only), irinotecan, simvastatin (erlotinib only), teriflunomide (gefitinib only), triazolam (erlotinib only), vemurafenib (gefitinib only), vinblastine, vincristine, vindesine, and vinorelbine
- Altered distribution and excretion of erlotinib or gefitinib: amiodarone (erlotinib only), azithromycin (erlotinib only), ciclosporin (erlotinib only), and vemurafenib (erlotinib only)

#### Secondary Variables

A secondary variable will be comorbidity. Comorbidity will be assessed using a previously validated pharmacy-based comorbidity index for patients with cancer [29] with further adaption for this study. Comorbid conditions will be identified from community pharmaceutical dispensing records for a 9-month period starting 1 year before the commencement of treatment with erlotinib or gefitinib. A total of 19 comorbid disorders will be identified according to the dispensing of ≥1 specific pharmaceuticals or those from specific therapeutic groups, as shown in Multimedia Appendix 3. The number of comorbid disorders for each patient will be summed without weighting. The validity of national electronic health databases for identifying comorbidities was assessed in the validation substudy (Multimedia Appendices 1 and 2).

Other secondary variables, which will be used in patient characterization and analyses of exploratory factors, covariates, and confounders, will include age, the date of diagnosis, the date of death, gender, ethnicity, the place of domicile, tumor morphology, the basis of diagnosis, notifying laboratory, the extent of disease, tumor stage, tumor grade, deprivation index, rurality, EGFR mutation subtype, EGFR test method, smoking status, clinical stage, and performance status.

### Statistical Analysis

The process of assembling the retrospective cohort will be illustrated using a flow diagram to show the number of patients at each stage of the process, including those who were examined for eligibility, were included in the study, completed follow up, were included in the analysis or not included, and the reasons for noninclusion. Descriptive statistics will be used to analyze the demographic profiles, clinical characteristics, and treatment received by the assembled retrospective cohort. To determine the effectiveness and safety of erlotinib and gefitinib in the cohort (aim 1), time to event effectiveness (time to discontinuation of treatment with erlotinib or gefitinib, overall survival, and lung cancer–specific survival) and safety outcome measures (non–cancer-related mortality) will be analyzed using Kaplan-Meier curves and Cox proportional hazards modeling to generate summary survival statistics (median, 1-year, and 2-year survival, as well as 95% CIs) and examine predictive factors. To identify and assess serious adverse events (aim 2), summary tables and descriptive statistics will be used to display the number and percentage of patients with serious adverse events by ICD-10 code and their known association with erlotinib or gefitinib. To determine the prevalence and patterns of concomitant medicines use (aim 3), summary tables and descriptive statistics will be used to display the number and percentage of patients administered medicines concomitantly by individual specific medicine, therapeutic class, and expected adverse outcome of potential interaction with erlotinib or gefitinib. To analyze associations between high-risk concomitant medicines use and the effectiveness and safety of erlotinib and gefitinib (aim 4), study participants will be stratified according to whether they were exposed or not exposed to high-risk concomitant medicines. Differences between participants exposed to high-risk concomitant medicines and those not exposed in proportions of patients experiencing serious adverse events will be analyzed by chi-square tests and multivariable regression models. Differences between participants exposed to high-risk concomitant medicines and those not exposed in time to event end points will be analyzed using Kaplan Meier curves and Cox proportional hazards modeling. Exploratory analyses may examine associations between the use of concomitant medicines and serious adverse events or poor survival outcomes using different definitions of concurrent exposure, individual medicines, the therapeutic classes of medicines, and patient subgroups such as those defined by ethnicity. Sensitivity analyses may be undertaken, for example, to assess effects on the outcomes of modifying patient eligibility criteria.

### Sample Size Considerations

With an expected cohort of between 800 and 900 eligible participants, with ≥750 events, the study will have 80% power (=.8) for detecting with statistical significance (=.05) differences in time to treatment discontinuation (primary effectiveness outcome measure) with hazard ratios of ≤0.76 between the groups, each including between 20% and 80% of the whole cohort. With an expected rate of serious adverse events (primary safety outcome measure) of ≥10% and complete serious adverse event data on at least 75% of the eligible cohort participants, the study will have 80% power (=.8) for detecting with statistical significance (=.05) differences of at least 2-fold in serious adverse event rates between the groups, each including between 20% and 80% of the whole cohort.

### Validation Substudy

To validate national electronic health databases as the source of data for this study protocol and the aforementioned methods for determining patient eligibility and identifying the key outcomes and variables of interest, the validation substudy was undertaken in a subgroup of potentially eligible patients. From a source population of 532 patients identified in national electronic dispensing records as having been dispensed erlotinib or gefitinib in the northern region of NZ between October 1, 2010, and September 30, 2020, a total of 100 (18.8%) patients were randomly selected for this validation substudy. The northern region includes the Auckland, Counties Manukau, Waitemata, and Northland district health areas and one-third of the national population. The validation substudy was restricted to northern region patients because their clinical records were available to the investigators for manual review. A gold standard set of data was compiled for each validation substudy patient by manual review of their clinical records, including their EGFR mutation test status. These results were compared to those generated by study methods applied to data compilations from national electronic health databases. As the primary cause of death was not systematically recorded in clinical records, fatal and nonfatal serious adverse events were validated separately. In addition, fatal serious adverse events were validated without the requirement of being non–cancer related and termed for this purpose as *potential serious adverse events that were fatal*. Categorical data were analyzed by agreement analysis and numerical data by paired 2-tailed *t* tests. A sample size of 100 patients for the validation substudy was expected to be adequate for defining levels of overall agreement with 95% CIs of –10% to +10%.

### Ethical Considerations

Ethics approval for this research was obtained from the New Zealand Ministry of Health Northern B Health and Disability Ethics Committee (13/NTB/165/AM02). All procedures will be conducted in accordance with the relevant ethics guidelines (eg, the Declaration of Helsinki). As this research will retrospectively analyze routinely collected data and not involve direct contact with patients, the human participants included in this retrospective cohort study will not be able, or required, to give informed consent. Requirement for informed consent was considered impractical and undesirable by the researchers, ethics committee, and governance groups who approved the study. Patient confidentiality will be maintained by holding any identifying information as password-protected data spreadsheets or as hard copy data in secure facilities only accessible to approved research or clinical personnel whose employment contracts include obligations regarding patient confidentiality.

## Results

### Validation of Categorical Data

Validation analyses for categorical data are shown in Multimedia Appendix 1. For the determination of patient eligibility, the comparison of proxy and standard eligibility criteria applied to national electronic health databases and clinical records, respectively, showed overall agreement and κ statistic of 96% and 0.8936, respectively. For the identification of nonfatal serious adverse events, the comparison of national electronic health databases and clinical records showed overall agreement and κ statistic of 93% and 0.8526, respectively. For the identification of potential serious adverse events that were fatal, the comparison of national electronic health databases and clinical records showed overall agreement and κ statistic of 100% and 1.0000, respectively. For the identification of interruptions, reductions, or substitutions of treatment with erlotinib or gefitinib, the comparison of national electronic health databases and clinical records showed overall agreement and κ statistic of 94% and 0.8764, respectively. For the identification of serious adverse drug reactions, the comparison of national electronic health databases and clinical records showed overall agreement and κ statistic of 97% and 0.8529, respectively. For the identification of the use of high-risk concomitant medicines with erlotinib or gefitinib (primary variable), the comparison of national electronic health databases and clinical records showed overall agreement and κ statistic of 95% and 0.8717, respectively. For identifying comorbidities, the comparison of national electronic health databases and clinical records showed overall agreement and κ statistic of 93% and 0.7779, respectively.

### Validation of Numerical Data

Validation analyses for numerical data are shown in Multimedia Appendix 2. To validate national electronic health databases as the source of key dates for determining time to discontinuation of treatment with erlotinib or gefitinib (primary effectiveness outcome) and overall survival, the dates of the first dispensing of erlotinib or gefitinib, last treatment, and death were compared to those in the clinical records. For the dates of the first dispensing of erlotinib or gefitinib, national electronic health databases and clinical records differed on average by approximately 4 days with a nonsignificant *P* value of .33 and CIs overlapping with zero difference. For the date of last treatment with erlotinib or gefitinib, national electronic health databases and clinical records differed on average by approximately 6 days with a nonsignificant *P* value of .46 and 95% CIs overlapping with zero difference. For the dates of death, national electronic health databases and clinical records differed on average by <1 day with a nonsignificant *P* value of .57 and 95% CIs overlapping with zero difference. For the number of comorbidities, national electronic health databases and clinical records differed on average by <1 comorbidity but with a marginally significant *P* value of .03 and 95% CIs almost overlapping with zero difference.

### Current Status

As of May 2024, the main study is ongoing.

## Discussion

### Summary

The research project described in this protocol will provide important new information about the everyday impacts, both beneficial and harmful, arising from the introduction of erlotinib and gefitinib into routine clinical use for treating advanced EGFR mutation–positive lung cancer in NZ a little more than 1 decade ago. Previous studies in this area have been limited to evaluations of the impact and uptake of the introduction of EGFR mutation testing in the northern region of NZ [4,30-32]. Currently, few data exist on the effectiveness and safety of erlotinib and gefitinib in the general NZ patient population and on the contributions made by concomitant medications and other factors to adverse outcomes in this setting of routine care. Such new real-world and NZ-specific data are likely to provide deeper insights into the harms profiles of erlotinib and gefitinib and to have important implications for clinical practice and patient-centered care; for example, if the study establishes associations between adverse outcomes and the use of concomitant medicines, an awareness of these data could influence the clinical practices of health care professionals who prescribe or dispense erlotinib, gefitinib, or other medicines for use concomitantly with erlotinib and gefitinib in NZ or elsewhere. The data will also inform new health policies, such as those concerning state subsidies for newer-generation EGFR kinase inhibitor drugs that have gained regulatory approvals for marketing but remain unfunded in NZ since erlotinib and gefitinib were introduced into routine care approximately 1 decade ago.

In the validation substudy, we showed that the methods proposed in the study protocol, when applied to data compiled from national electronic health databases, were valid for determining patient eligibility and identifying the outcomes and variables of interest to this retrospective cohort study. Compared to gold standard data sets compiled by a manual review of clinical records, the results generated by the study methods applied to data compiled from national electronic health databases showed good levels of agreement in categorical data, such as patient eligibility, serious adverse events, and the use of high-risk concomitant medicines, and generally small and statistically insignificant differences in numerical data such as the dates required for the estimation of time to treatment discontinuation. These results support the validity of the methods described in the study protocol and the robustness of the findings that will be generated and eventually reported.

### Strengths

The strengths of the research protocol include its large population-based sample, internal validity, national generalizability, and the unique patient cohort. We envisage recruiting almost all eligible patients from throughout NZ in a whole-of-patient-population cohort, thereby eliminating selection biases and assembling one of the largest national cohorts of this type to be reported to date [33-37]. However, few of these previous studies have evaluated serious adverse events from erlotinib and gefitinib and their association with the use of concomitant medication in real-world settings. The use of national electronic health databases that use consistent data collection methodology will give confidence that the study results will be generalizable to all NZ patients and regions. Previous studies have highlighted striking geographic and ethnic variations in EGFR mutation–positive lung cancer [38,39]. As NZ is a multiethnic society, with the largest global populations of Indigenous Māori and Pacific people, this study will provide important new information of international interest about treatment outcomes in these understudied groups.

### Limitations

The limitations of the study protocol include potential biases from errors inherent in the use of routinely collected data; for example, the safety outcomes for the study protocol assume that the coding of hospital admissions and deaths is always complete and accurate, whereas this is not necessarily the case. Further limitations of the retrospective study design include that, unlike in prospective randomized studies, it will not be possible to assess causality or control for confounding variables. The study design also assumes adherence to the treatment regimens as recorded in dispensing records and provides no practical method for assessing compliance. Another limitation is the historical perspective of the study protocol, which will evaluate the impacts of treatment that occurred between 2 and 12 years ago and may now be of limited relevance to current clinical practice. For the initial assessment of patient eligibility, the study protocol infers EGFR mutation status indirectly from pharmaceutical dispensing records rather than directly from EGFR mutation test results, which could have introduced bias. The findings of the study may not be generalizable outside NZ because of limited external validity and a shift in standard EGFR treatments to targeted therapies that have superseded gefitinib and erlotinib. However, if the study establishes associations between adverse outcomes and the use of concomitant medicines, these findings could have important implications for the use of erlotinib or gefitinib in other countries or for the use of other anticancer kinase inhibitor drugs susceptible to similar medicine interactions [40]. The limitations of the validation study included its small sample size and the resulting wide CIs for the levels of agreement, κ statistic, and other statistical parameters.

### Conclusions

In conclusion, a protocol is presented for a planned study that seeks to describe the safety and effectiveness of erlotinib and gefitinib during the first decade of their routine clinical use in NZ for treating EGFR mutation–positive lung cancer in a national whole-of-patient-population retrospective cohort. An exploratory substudy has demonstrated the feasibility and validity of using national electronic health databases and the study methodology developed for determining patient eligibility and identifying the outcomes and variables of interest for this study.

## Appendix

Multimedia Appendix 1 Validation of national electronic health databases as the source of categorical data for a national retrospective cohort study of the safety and effectiveness of erlotinib and gefitinib for treating advanced epidermal growth factor receptor mutation—positive lung cancer in Aotearoa New Zealand.

Multimedia Appendix 2 Validation of national electronic health databases as the source of numerical data for a national retrospective cohort study of the safety and effectiveness of erlotinib and gefitinib for treating advanced epidermal growth factor receptor mutation—positive lung cancer in Aotearoa New Zealand.

Multimedia Appendix 3 Comorbidity definitions by pharmaceutical dispensing for a national retrospective cohort study of the safety and effectiveness of erlotinib and gefitinib for treating advanced epidermal growth factor receptor mutation-positive lung cancer in Aotearoa New Zealand. Italicized names and ID numbers to be used in searches of dispensing records.

## Abbreviations

EGFR: epidermal growth factor receptor
ICD-10: International Classification of Diseases, Tenth Revision
NZ: Aotearoa New Zealand

## Data Availability

All aggregate data (ie, counts) required for replicating the calculation of proportions, and so on, will be made available within manuscripts. Individual patient-level data cannot be shared publicly because they will contain identifying patient information. Ethics and legal requirements, which limit data access and sharing to maintain patient confidentiality and privacy, have been imposed upon us by the New Zealand Ministry of Health ethics and governance committees.
