# Supplementary material for: Erlotinib or Gefitinib for Treating Advanced Epidermal Growth Factor Receptor Mutation–Positive Lung Cancer in Aotearoa New Zealand: Protocol for a National Whole-of-Patient-Population Retrospective Cohort Study and Results of a Validation Substudy
Source: JMIR Res Protoc. 2024 Jul 2;13:e51381. doi: 10.2196/51381 (PMC11252616; doi:10.2196/51381)
Supplement: Multimedia Appendix 1 [file resprot_v13i1e51381_app1.pdf]

Study design		National electronic health databases	Clinical records			Agreement analysis	
			No	Yes	Total	Overall agreement	Kappa Statistic
Eligibility		No	23	4	27	96%	0.8936
		Yes	0	73	73		
		Total	23	77	100		
Primary Safety Outcome	Nonfatal Serious Adverse Events	No	58	1	59	93%	0.8526
		Yes	6	35	41		
		Total	64	36	100		
	Potential Serious Adverse Events – Fatal	No	74	0	74	100%	1.000
		Yes	0	26	26		
		Total	74	26	100		
Secondary Safety Outcomes	Interruption, reduction or substitution of erlotinib or gefitinib	No	56	6	62	94%	0.8764
		Yes	0	38	38		
		Total	56	44	100		
	Serious adverse drug reactions	No	87	3	90	97%	0.8529
		Yes	0	10	10		
		Total	87	13	100		
Primary variable	High-risk concomitant medicines use	No	24	3	27	95%	0.8717
		Yes	2	71	73		
		Total	26	74	100		
Secondary variable	Comorbidity	No	16	1	17	93%	0.7779
		Yes	6	77	83		
		Total	22	78	100		
