# Supplementary material for: Erlotinib or Gefitinib for Treating Advanced Epidermal Growth Factor Receptor Mutation–Positive Lung Cancer in Aotearoa New Zealand: Protocol for a National Whole-of-Patient-Population Retrospective Cohort Study and Results of a Validation Substudy
Source: JMIR Res Protoc. 2024 Jul 2;13:e51381. doi: 10.2196/51381 (PMC11252616; doi:10.2196/51381)
Supplement: Multimedia Appendix 2 [file resprot_v13i1e51381_app2.pdf]

| Study design | | n | | Mean | Range | Pairwise differences | | |
|---|---|---|---|---|---|---|---|---|
| | | | | | | Mean | 95% CI | *P* |
| Primary effectiveness outcome - time to treatment discontinuation | Date of first dispensing of erlotinib or gefitinib | 100 | National electronic health databases | 5 Apr 2015 | 18 Oct 2010 to 4 Dec 2019 | 4.1 | -4.2 to 12.3 | 0.328 |
| | | | Clinical records | 9 Apr 2015 | 18 Oct 2010 to 4 Dec 2019 | | | |
| | Date of last treatment | 100 | National electronic health databases | 19 May 2016 | 14 Jan 2011 to 13 Jun 2021 | 6.3 | -10.4 to 23.0 | 0.455 |
| | | | Clinical records | 25 May 2016 | 14 Jan 2011 to 13 Jun 2021 | | | |
| Secondary effectiveness outcome – overall survival | Date of death | 84 | National electronic health databases | 30 Jun 2016 | 23 Jan 2011 to 24 Dec 2021 | 0.01 | -0.03 to 0.05 | 0.567 |
| | | | Clinical records | 30 Jun 2016 | 23 Jan 2011 to 24 Dec 2021 | | | |
| Secondary variable – comorbidity | Number of Comorbidities | 100 | National electronic health databases | 2.69 | 0 to 10 | -0.31 | -0.59 to -0.03 | 0.0288 |
| | | | Clinical records | 2.38 | 0 to 6 | | | |
