# Supplementary material for: Erlotinib or Gefitinib for Treating Advanced Epidermal Growth Factor Receptor Mutation–Positive Lung Cancer in Aotearoa New Zealand: Protocol for a National Whole-of-Patient-Population Retrospective Cohort Study and Results of a Validation Substudy
Source: JMIR Res Protoc. 2024 Jul 2;13:e51381. doi: 10.2196/51381 (PMC11252616; doi:10.2196/51381)
Supplement: Multimedia Appendix 3 [file resprot_v13i1e51381_app3.pdf]

Comorbidity	Therapeutic group 1 (ID)	Therapeutic group 2 (ID)	Therapeutic group 3 (ID)	Chemical (Chem ID)	Exclusions
Anaemia	Alimentary tract and metabolism (1)	Vitamins (137)	Vitamin B (13704)	*Hydroxocobalamin (1624)*	Not other vitamin B preparations
		Minerals (138)	*Iron (13830)*	Any	
	Blood and blood forming organs (4)	*Antianaemics (401)*	Any	Any	
Anticoagulation	Blood and blood forming organs (4)	Antithrombotic agents (407)	*Oral anticoagulants (40707)*	Any	
			Heparin and antagonist preparations (40704)	*Dalterparen sodium (3976); Enoxaparin sodium (3893); Heparin sodium (1589)*	Not heparinised saline (6019)
Antiplatelet	Blood and blood forming organs (4)	Antithrombotic agents (407)	*Antiplatelet agents (40701)*	Any	
Anxiety and tension	Nervous system (22)	*Anxiolytics (2225)*	Any	Any	Not sedative/hypnotics or barbiturates
Cardiac arrhythmias	Cardiovascular system (7)	Antiarrhythmics (713)	Antiarrhythmics (71301)	*Digoxin (1412); Amiodarone (1057); Disopyramide (1428); Flecainide (1510); Mexiletine (1823); Propafenone (2810)*	Not Atropine (1097) or propantheline (2861)
Congestive heart failure	Cardiovascular system (7)	Diuretics (731)	*Loop diuretics (73101)*	Any	
Depression	Nervous system (22)	*Antidepressants (2205)*	Any	Any	
Diabetes	Alimentary tract and metabolism (1)	Diabetes (113)	*Alpha Glucosidase inhibitors (11311)*	Any	Not Hyperglycaemic agents (11301) or Diabetes management (115)
			GLP-1 Agonists (11315)	Any	
			Insulin immediate acting preparations (11305)	Any	
			Insulin long acting preparations (11307)	Any	
			Insulin rapid acting preparations (11309)	Any	
			Insulin short acting preparations (11303)	any	
			Oral hypoglycaemic agents (11312)	Any	
Epilepsy	Nervous system (22)	Anti-epilepsy (2207)	*Control of epilepsy (220702)*	Any	Not Agents for control of status

Comorbidity	Therapeutic group 1 (ID)	Therapeutic group 2 (ID)	Therapeutic group 3 (ID)	Chemical (Chem ID)	Exclusions
					epilepticus (220701)
Gastric Acid disorder	Alimentary tract and metabolism (1)	Antiulcerants (110)	*Antisecretory and cytoprotective (11001)*	Any	Not Helicobacter Pylori Eradication (11002)
			H2 antagonists (11003)	Any	
			Proton Pump Inhibitors (11010)	Any	
			Site protective agents (11013)	Any	
Hyperlipidaemia	Cardiovascular System (7)	*Lipid modifying agents (732)*	Any	Any	
Hypothyroidism	Hormone preparations – systemic excluding contraceptive hormones (14)	Thyroid and antithyroid agents (1414)	Thyroid and antithyroid agents (141401)	*Levothyroxine (2263)*	Not carbimazole (1221) or propylthiouracil (2062)
Ischaemic Heart Disease -Angina	Cardiovascular System (7)	*Nitrates (734)*	Any	Any	
Ischaemic Heart Disease - Hypertension	Cardiovascular System (7)	*Agents Affecting the Renin Angiotensin System (707)*	Any	Any	
		Beta-adrenoceptor Blockers (716)	Any	Any	
		Calcium channel blockers (722)	Any	Any	
		Centrally acting agents (728)	Any	Any	
		Diuretics (731)	*Potassium sparing diuretics (73104)*	Any	
			Potassium sparing combination diuretics (73107)	Any	
			Thiazide and related diuretics (73110)	Any	
		Vasodilators (740)	Vasodilators (74001)	*Hydralazine (1604); minoxidil (2451)*	Not other vasodilators Abrisetam (3904); Amyl nitrate (6601); Bosentan (3889); Epoprostenil (4093); Iloprost (3891); Nicorandil (3975); Papaverine (1928); Pentoxifylline (2455); sildenafil (3890)

Comorbidity	Therapeutic group 1 (ID)	Therapeutic group 2 (ID)	Therapeutic group 3 (ID)	Chemical (Chem ID)	Exclusions
Parkinsons disease	Nervous system (22)	Agents for Parkinsonism and related disorders (2201)	*Anticholinergics (220104)*	Any	Not Agents for Essential Tremor, Chorea and Related Disorders (220107)
			Dopamine receptor agonists and related agents (220101)	Any	
Osteoporosis/ Pagets disease	Musculoskeletal system (19)	*Drugs affecting bone metabolism (1908)*	Any	Any	
Psychotic Illness	Nervous system (22)	*Antipsychotics (2222)*	Any	Any	
Reactive airways disease	Respiratory system and allergies (28)	*Anticholinergic agents (2834)*	Any	Any	
		Beta adrenoceptor agonists (2830)	Any	Any	
		Inhaled corticosteroids (2810)	Any	Any	
		Inhaled on-acting Beta adrenoceptor agonists (2824)	Any	Any	
		Leukotriene Receptor Antagonists (2837)	Any	Any	
		Methylxanthines (2843)	Any	Any	
		Mast cell stabilisers (2840)	Any	Any	
Steroid responsive conditions	Hormone preparations – systemic excluding contraceptive hormones (14)	Corticosteroids and related agents for systemic use (1407)	Corticosteroids and related agents for systemic use (140701)	*Betamethasone (2864); Dexamethasone (1383, 1385); Hydrocortisone (1617); Methylprednisolone (1810, 1811, 1812, 1816); Prednisone (2038); Triamcinolone (2389)*	Not Fludrocortisone (1515), Tetracosactrin (2389); inhaled corticosteroid (2810), nasal preparations (2851), Corticosteroids topical (1011); Psoriasis and Eczema preparations (1019); Scalp preparations (1022); Eye preparations (3103); Ear preparations (3101)
